# The Berlin reformed curriculum in undergraduate medical education: a retrospective of the development history, principles, and termination

**DOI:** 10.3205/zma001270

**Published:** 2019-10-15

**Authors:** Jutta Begenau, Claudia Kiessling

**Affiliations:** 1 Charité – Universitätsmedizin Berlin, Berlin, Germany; 2 Universität Witten/Herdecke, Fakultät für Gesundheit, Lehrstuhl für die Ausbildung personaler und interpersonaler Kompetenzen im Gesundheitswesen, Witten, Germany

**Keywords:** undergraduate medical education, reformed medical curriculum, problem-based learning

## Abstract

The Reformed Medical Curriculum (RMC) at Charité-Universitätsmedizin Berlin was launched in autumn 1999, while medical schools in Canada, the United States, Scotland, the Netherlands, and Scandinavia already had adapted educational reforms in medical education many years before [1], [2]. For eleven years, 63 medical students per year trained at the Faculty in accordance with international standards governing the RMC. It was the first and perhaps most revolutionary reformed medical curriculum at a German university after the commencement of “Modellklausel”, a new section in the Licensing Regulations for Doctors (Approbationsordnung für Ärztinnen und Ärzte) in 1999 that paved the way for fundamental reforms within undergraduate medical education in Germany. The idea was to establish and test a “pilot project of a fundamental reform of medical education in Germany” [3], thus aligning Germany with international developments and establishing a model for other reform initiatives. The first part of the article will provide an overview of how the RMC were able to emerge. It reports who initiated the project and why, who kept it running and encountered opposition and what were the social and political conditions. The second part of the article describes the principles that were fundamental for the development of the RMC. The third part illustrates the quality assurance measures, and the final section covers the termination of the RMC.

## Zusammenfassung

Der Reformstudiengang Medizin (RSM) an der Charité-Universitätsmedizin Berlin wurde im Wintersemester 1999/2000 eröffnet, zu einer Zeit, als medizinische Fakultäten in Kanada, den USA, Schottland oder Skandinavien bereits viele Jahre nach reformierten Ausbildungskonzepten unterrichteten [1], [2]. An ihm wurden – insgesamt elf Jahre lang – jährlich 63 Studierende immatrikuliert, um an einem nach internationalen Standards ausgerichteten Curriculum ausgebildet zu werden. Er war der erste Reformstudiengang nach Modellstudiengangsklausel an den deutschen medizinischen Fakultäten und vielleicht auch der revolutionärste. Als „Pilotprojekt zur Erprobung einer grundsätzlichen Reform des Medizinstudiums in der Bundesrepublik Deutschland“ [3] gedacht, ermöglichte er zum einen die Annäherung Deutschlands an die internationalen Entwicklungen, zum anderen wurde er das Modell für viele der folgenden Reformansätze. Wie der RSM entstehen konnte, wer diese Revolution und warum initiierte, wer sie am Laufen hielt und dabei auch auf Gegnerschaft stieß und unter welchen sozialen und politischen Rahmenbedingungen sich dies abspielte, davon wird im ersten Teil des Beitrages die Rede sein. Gegenstand des zweiten Teils sind die Grundsätze, nach denen das Curriculum entwickelt wurde. Der dritte Teil beschäftigt sich mit der Sicherung der Qualität des RSM. Den Abschluss bildet der vierte Teil mit dem Ende des RSM.

## 1. From a student strike to the launch of the reformed medical curriculum in Berlin

The following section draws from several sources and five interviews with two former student activists and three medical doctors who became reform activists at an early stage: Prof. Dr. med. Walter Burger, head of the RMC working group from 1995 to 2005 (interview partner 1 WB); Prof. Dr. med. Joachim Dudenhausen, Dean of the Charité from 2001 to 2004 and longstanding head of the RMC Study Committee (interview partner 2 JD); Prof. Dr. med. Claudia Kiessling, co-founder and member of the RMC working group (interview partner 3 CK); Dr. med. Udo Schagen, head of the research unit for Contemporary History at the Institute of History in Medicine and one of the first supporters of the RMC (interview partner 4 US); and Dr. med. Kai Schnabel, co-founder and member of the RMC working group (interview partner 5 KS). 

The birth of the RMC is marked by the students’ Uni-Mut strike in autumn 1988/89 [4], [5], [6], [7]. The strike started at the Free University Berlin (FUB), soon spread across the entire West Germany, and lasted nearly a full semester. Students were dissatisfied with their study conditions and lack of academic and political participation, for example with regard to the ongoing restructuring measures of departments and institutes at the FUB. What all participants in the strike had in common was the experience of studying at a mass university [6]. However, the medical curriculum was problematic as well. “The course of study was not really scholarly to my mind. Every subject was covered four times: in a lecture, in a laboratory course, in a seminar and in a tutorial. In addition, it sometimes came up in an introduction into the lab course. (…) That was not how I imagined the study of science. Self-directed learning was out of question, it was completely directed by others” [KS, S.10]. The student strikers outlined the initial ideas regarding study reforms in an eight pages long paper, called “Berlin Model” and passed it in a plenary assembly attended by more than 1,000 students in late 1988 [[8], CK]. However, reforms needed legal regulations and supporters. 

### 1.1. Initiators, supporters, opponents, and circumstances 

The initiators were students who expressed dissatisfaction with their medical education during the Uni-Mut strike in 1988/89. Following the strike, a group of students continued to work on reform ideas and showed considerable stamina. What united them was the belief that medical education that focuses on self-directed, exemplary, patient-oriented, and practice-oriented learning would prepare them better for their professional career than the traditional course of study. 

The ten medical students who kept going after the strike were lucky and proved to possess persuasive power. They succeeded in convincing decision makers within the faculty to support them and push the process forward [WB].Those were Rolf Winau, Udo Schagen and Eberhard Göbel from the Institute of History of Medicine, Dean Dieter Scheffner and Vice Dean Joachim Dudenhausen from the University Hospital Rudolf Virchow, paediatrician Walter Burger, and later on – after the merging of two medical faculties – both Deans, namely Harald Mau and Joachim Dudenhausen, and numerous colleagues from the Charité with a strong awareness of and interest in teaching and learning [JD, S.8]. 

However, there were also more or less open opponents. They were lacking – like many other university teachers elsewhere [9] – the “understanding of the necessity to reform medical education” [9]. One of their reservations was: by focusing on practical applications and skills, the planned reform would thus weaken the scientific foundation and produce “barefoot doctors”. Among them were also teachers who defended their “privileges” [JD, S.7] and who believed that their “wonderful lecture was the most meaningful they could provide” [JD, S.7]. 

Based at the FUB, the opponents were mainly located at preclinical departments in Berlin Dahlem and were known to be “among the most conservative ones in Germany” [KS, S. 24]. One of the activists remembers an “incredible obstructionism from the Free University” [WB, S.7]. 

The reform efforts were supported by a nationwide debate about education reforms, which started in the seventies and picked up pace in the eighties [10], [11], [12]. The debate acknowledged that “evidence-based expertise had developed internationally” [11] regarding necessary changes in medical education and that many internationally renowned medical faculties in the United States, Canada etc. had already started to implement reforms. 

Prominent reform supporters included members of the Murrhardter Kreis – founded by the Robert Bosch Foundation – and members of the Carl Gustav Carus Foundation, namely Thure von Uexküll [13] und Hannes Pauli [14]. Vital impulses came from the Science Council (Wissenschaftsrat) [15] and a Council of Experts (Sachverständigenrat) implemented by the German Ministry of Health [16]. Furthermore, students were supported by experts from well-known reform universities like Maastricht, Hamilton (McMaster), Albuquerque, and Linköping. 

#### 1.2. Start, crisis, and implementation: challenges 

This section will focus on ten years of developing the RMC. According to the interviews, the history of the RMC can be subdivided in three phases with different challenges and tasks. 

##### 1.2.1. Start: „Many things have been worked out“ 

Starting point was the “torso” of the “Inhalts AG”, the group that was founded during the strike to discuss study conditions, and the ten students who kept the group alive after the strike. They “pushed the plans forward to implement the Berlin reform model“ [17], initially “via politics (…), Hilde Schramm, a prominent parliamentarian of the Green Party – the Berlin Senate was governed by SPD and Greens –and there we were welcomed with open arms” [KS, S.20]. And indeed, the Berlin Parliament approved extra funding for student-led university projects (Projekttutorien) in summer 1989, which made it possible for students to apply for funding [[6], US, S.4]. Members of the Inhalts-AG found their first home at the Institute of History of Medicine, at the Research Unit for Contemporary History. They established a reading circle that addressed theories of medicine, the biopsychosocial model, and the ideas of Thure von Uexküll [18]. 

A first workshop “Reformed Medical Education – Content, Structure, and Steps towards Realisation” (Medizinischer Reformstudiengang – Inhalt, Struktur und Schritte zu seiner Verwirklichung) was implemented in November 1989 with approximately 300 participants [6], [19], [20]. It prepared “the ground for financing the Planning Group RMC (PlaGru RMC) through a special programme of the Federal Land of Berlin” [6]. 

In March 1990, PlaGru RMC began operation with four scientific and several student employees [20]. Head of the group was Dieter Scheffner, Dean of the University Hospital Rudolf Virchow, supported by his Vice Dean Joachim Dudenhausen. At the same time, medical doctors at the University Hospital Westend started to compile a catalogue of learning objectives. Dudenhausen recalls: “that must have been in winter 1989/90.” In addition, considerable efforts were undertaken to convince the faculty “in the background, not in secret, but not as part of daily routine. Knocking on colleagues doors.” [JD, S. 7]

PlaGru RMC, together with the still existing Inhalts-Ag, dedicated itself to organising workshops and conferences as well as short-term courses with innovative educational formats. In early January 1991, a second workshop “New Learning and Teaching Methods in Medical Education” (Neue Lern-und Lehrformen im Medizinstudium) took place [21]. A third workshop called Loccum Conference in July 1992 attracted international facilitators and participants and dealt with the topic Changing Medical Education [22]. With the intent to learn from others, members of PlaGru and Inhalts-Ag visited the University Witten/Herdecke (Germany), McMaster University (Canada), the University of New Mexico (USA), Maastricht University (the Netherlands), Scandinavian reform universities in Tromsö and Linköping, and the University in Berne (Switzerland) [2]. A first draft of a medical education reform (Berlin Model Curriculum) based on these experiences was submitted for evaluation at the Scientific Council in May 1992 [20]. Then, the momentum was temporarily lost. The Berlin Model Curriculum was under evaluation with the Scientific Council. Responsibilities were not clear. That situation led to a crisis and “the contracts (of the reform group) were not extended” [JD, S.10] 

##### 1.2.2. Crisis, restart, and stagnation 1993 to 1996 

The engine of the movement stuttered but it did not stop. Scheffner initiated a “second start” [CK, S.10]. “Walter Burger came on board. He was the first new scientific member of the group. Mrs. Gregor became secretary and then somehow things got better” [CK, S.10]. Walter Burger recalls when Scheffer approached him to secure his support with the words: “You know, Mr. Burger, everything has been worked out, at McMaster and so on. The only thing we have to do now is to adapt it to Berlin” [WB, S. 6-7]. However, the situation was more complicated. The new team discovered, that “it was difficult to simply take things over. Instead, everything needs to be developed from scratch” [WB, S.7]. Accordingly, “we started with Adam and Eve, with thousand things, with statistics about morbidities and their representation in medical education and endless discussions” [WB, S.7].

In 1993, a Coordination Council (KoRa) was founded to promote the involvement of the FUB Medical Faculty into the development of the RMC and to “familiarise the faculty at an early stage” with novel learning methods such as problem-based learning (pbl) [23]. However, not everybody who was asked to participate was enthusiastic about it. The professor of anatomy for example (…) resigned in a “very theatrical manner” [WB, S.7]. His explanation was that “taking part in such a curriculum could not be squared with his conscience” [WB, S.7]. A pharmacologist expressed his opinion that all this “eco and psycho nonsense” was dispensable, and that “nurses and suchlike professions” were in charge of “pastoral work” [24]. It came to “fateful meetings”, work was marked by “standstill and intrigues”, and it became necessary to hold informal talks (“fireside chats”) again and again. Against all odds, the so-called “white folder”, the “Bible of the RMC” [KS, S.28] was developed. It contained educational objectives, contents, and diseases that required treatment. The latter were selected in interdisciplinary groups according to their exemplary character (important to understand basic processes and principles), urgency (essential action competence), and frequency [25].

In the meantime, the merging of the medical faculties at Humboldt University and the Free University was in full progress. For educational reformers, the year 1995 brought stagnation once again. “It took a year to draw up the legal requirements to detach the University Hospital Rudolf Virchow from the Free University and incorporate it into Humboldt University (…). That year was marked by merging and fights against and for and together and so on. There was no time for other things” [JD, S.7-8]. The merging process itself proved positive for educational reformers, because many teacher at the Charité, the medical school at Humboldt University, viewed “Scheffners reform ideas” favourably [[4] JD, S.7-8]. 

##### 1.2.3. Planning the implementation 1996 to 1999

In 1996, work priorities changed. Now, financing, faculty development, and fine-tuning of the curriculum and teaching methods became more and more important. The planning group PlaGru was renamed in working group (AG RMC) to reflect these new priorities. Walter Burger remained head of the group. Four and later five academic employees were recruited, among them reformers from the first generation. The planning of the implementation started. “Faculty development became a major issue” [JD, S.10]. A number of working groups was established to plan modules together with representatives of individual disciplines. The “white folder” was completed. To create a pool of future teachers, legal requirements for habilitation (a German postdoctoral qualification) were changed. The new rules stipulated that everybody who wanted to take that qualification must hold specific seminars [JD, S.10].

Another issue was financing. It became clear that the RMC would not be financed by the Medical Faculty or University (e.g. funds for members of the working group or infrastructure). Therefore, it was necessary to find other financial sources. “The primary funding was provided by the Robert Bosch Foundation, as far as I remember, together with the Conference of Education Ministers (…) If they had not financed it, Berlin would not have financed it either” [JD, S.11] 

In addition to the Berlin Senate, Volkswagen Foundation, Bund-Länder Commission for Educational Planning and Research Promotion, and Carl Gustav Carus Foundation agreed to fund the RMC. They all facilitated the implementation of the RMC. However, the project was still touch and go, as evidenced by repeated short-term contracts for the staff. 

In February 1999, the Licensing Regulations for Doctors were amended for the eighth time, and a new paragraph, the so-called “Modellklausel”, allowed trial projects of education reforms. This provided the legal framework to run reformed medical curricula, including the Berlin RMC. Just a month earlier, the AG RMC had submitted a draft to the Faculty Board to establish a “Study Committee for the preparation of the RMC” [26]. Approved by the faculty, Joachim Dudenhausen became head of the Study Committee. 

In autumn 1999, the time had come. The first 63 medical students enrolled in the RMC, which were run side by side with the traditional curriculum. The small number of students was due to evaluation issues. Three seminar groups with 21 students each and nine pbl/communication skills groups with seven students each made up the annual cohort. Students were randomly selected among candidates admitted at the Charité who volunteered to attend the RMC. The faculty was thus able to compare the different student groups in accordance with a non-inferiority trial [27], [28], [29]. “Everyone felt immensely optimistic. We (the AG RMC) learnt a lot during that time.” [WB, S.14]

At first, the first RMC students were quite irritated. One of the reform activists recalls that “students were of course completely unsettled. They were asking ‘will we be able to learn anything at all?’ The anatomists played an inglorious role once again by gleefully stating that the students would never become sound physicians with a course of study lacking any decent dissection course” [WB, S.14]. In the first weeks, they would cancel scheduled courses, because additional staff hadn’t been allocated yet. Several interventions by Dean Joachim Dudenhausen were necessary. The beginning required enormous effort, but eventually things settled down and academic work and medical education commenced. 

## 2. Implementation: principles of structure, content and educational methods, or: what made it revolutionary

The RMC was characterised by a number of principles that governed its design and implementation [30], [31]. The following section will highlight the most important of these principles.

- The future doctor: biopsychosocial model and patient-centred medicine
- Case-based and integrated learning
- From teaching to learning: student-centred education
- From content to objectives: constructive alignment via educational objectives and decluttering the curriculum
- Shared planning and decision making: from professor to interdisciplinary planning groups
- Learning from others: implementing evidence-based education and establishing medical education research

### 2.1. The future doctor: biopsychosocial model and patient-centred medicine

The demand for a “different kind of medicine” had been present from the very beginning of RSM development. The movement was spearheaded by Thure von Uexküll, Hannes Pauli, Robert Wiedersheim [32] and the “Murrhardter Kreis”, who supported and supervised the ideas and development of the RMC from the start. The aim was to broaden traditional biomedical medicine by psychosocial aspects and the patient perspective to epistemically consolidate medicine. The participants’ own experience with an unsatisfactory healthcare system, coupled with an analysis described by the Murrhardter Kreis as “crisis of medicine” had both given rise to that demand. That crisis was characterised by increased specialisation in the medical profession, demographic changes, a rapid increase in medical know-how, and an increasing technologisation of medicine. All these aspects were linked with specific bioethical challenges and a change of the qualification profile for future doctors [[1], S.59f]. 

As far as medical education was concerned, there was a rising demand for an academic discussion of the epistemological foundation of medicine at an early stage of the degree course and the option to explore the subject from interdisciplinary perspectives (e.g. in seminars about principles of medical theory and practice, and extracurricular studies in so-called Studium Generale), early patient contact and practical approach (in particular in primary care, implemented as early internships in private practices), as well as the implementation of a longitudinal curriculum of communication and social competencies. 

#### 2.2. Case-based and integrated learning

A change of perspective was the aim, characterised from sign to symptom (from diagnosis to subjective illness), in order to teach patient perspective. The implementation of problem-based learning as the central learning method was one way to emphasise that change of perspective. In the process, students started with individual symptoms exhibited by a patient (case-based learning), thus acquiring basic and clinical knowledge, clinical reasoning skills, and the skills necessary for the generation of integrated treatment plans, depending on the stage of their degree course. That learning process was complemented by interdisciplinary seminars, clinical skills training, practical and lab courses. No lectures were scheduled in the beginning at all. Occasional lectures providing a first overview over a new topic or discipline were implemented at a later stage following the students’ requests. Seminars were always held by two teachers, e.g. one from basic science and one from a clinical discipline. The idea behind that approach was to overcome the separation of preclinical and clinical disciplines and to demonstrate the interdisciplinary character of medical work. 

#### 2.3. From teaching to learning: student-centred education

As the RMC had originated in a student strike, students’ autonomy and self-determination was another major issue. At the beginning (early 1990s), that meant for example that teachers from the faculty were not welcome as group facilitators in many of the self-organised pbl groups, because they would inhibit the students’ learning process. Later on, this radical position was modified as experience and expertise with pbl increased, and the role of teachers was redefined. They became “midwives” or “catalysts” for the learning process. Teaching and learning changed from a teacher-centred to a student-centred approach. This constituted a drastic change of medical culture and a major reason of opposition and resistance on the part of more conservative faculty members. That development was perceived as a loss of control over the learning process on the one hand, and on the other hand as a shift in the attitude towards students: from ignorant children to self-determined adults in charge of their own life. In view of the above, it was inevitable for pbl to be implemented as the central learning method. 

Participation in seminars, clinical skills training, and laboratory courses was voluntary to give students the opportunity to decide for themselves in what way they would approach their educational objectives. It soon emerged that students regularly attended classroom events, because this was where educational objectives were addressed and where enabled students had the chance to discuss questions with experts. 

In addition to pre-structured classroom events, the timetable provided for plenty of time for self-study. The necessary infrastructure was established to support self-study (development of the first skills lab in German-speaking countries – the training centre for clinical skills TÄF – and the expansion of the library), and courses about “learning to learn” were included into the first-semester curriculum. Students were able to choose individual elective subjects (clinical electives, research electives, seminars about principles of medical theory, Studium Generale). The only compulsory courses were the ones that required teamwork and focussed on competencies that could not easily be assessed at that time: pbl, internship in private practices, work placements on hospital wards, and communication skills trainings, i.e. courses that were always linked with reflection (e.g. patient contacts and experience in clinical settings). In the latter, simulated patients have been deployed since the year 2000. 

#### 2.4. From content to objectives: constructive alignment via educational objectives and decluttering the curriculum

Case-based and problem-based learning in connection with substantial time allocated for self-study resulted in a significant decrease of pre-structured teaching (e.g. the abolition of lectures, the abolition of the dissection course) and in a painful decluttering of teaching content for many teachers. Learning was no longer based on systematics (e.g. in physiology or internal medicine), but became exemplary. Learning content was selected in accordance with the requirements of residents working in the field of primary care. “It was not possible to explain the entire canon of medicine. Apart from that, knowledge is growing old too fast… Students just have accept that they can’t ever know everything and learn to recognise what is important for a general practitioner, a qualified general practitioner. This is what you should teach” [JD, S.13]. 

The development of organ or topic-based modules was based on educational objectives, which were subdivided into cognitive, applied, and affective objectives. The learning spiral with recurrent topics and increasing complexity facilitated cumulative learning. Educational objectives governed the scheduled, taught, learned, and assessed curriculum. Examinations assessed the objectives of the modules and not the content of classroom events. Examinations were interdisciplinary and limited to one written and one practical examination in each semester [33].

#### 2.5. Shared planning and decision making: from professor to interdisciplinary planning groups

Another basic principle was the planning and decision-making pathway. The requirement was to establish collaborative work among different disciplines and status groups. Objectives and contents of individual modules were discussed in interdisciplinary groups, rather than being determined by single representatives of single disciplines. Many professors had to get used to that approach. Some professors considered it outrageous that students were invited as co-decision-makers. This approach aimed at involving everybody in the process of developing and implementing the new curriculum, and at establishing commitment and ownership, i.e. specifically applying principles of change management [34], [35]. The highest decision-making body was the Study Committee where all plans for the new modules were discussed and approved. Many RMC staff members followed a “philosophy of the open door” to signal that everybody – teachers and students alike – was invited to come in, to give positive or negative feedback, or to just say “hello”. Establishing and running all workings groups for modules and longitudinal courses required much effort, but it eventually constituted the foundation for personal interaction and exchange among colleagues. Moreover, it gave birth to small-scale medical education research projects, initiated by interested teachers who started to evaluate parts of the curriculum with the help of the AG RMC, partly by comparing outcomes of these parts with traditional medical curriculum that existed side by side with the reformed curriculum [e.g. [36], [37], [38], [39], [40]].

#### 2.6. Learning from others: implementing evidence-based education and establishing medical education research

Many of these projects were realised because the innovative educational approaches could be tested with the relatively small cohorts. Ever since the RMC was launched, the reformers in Berlin benefited from the outstanding generosity of distinguished experts. Some of them spent a sabbatical in Berlin (e.g. Scott Obenshain from Albuquerque, Robert Wiedersheim from Witten). Some came for a week and supported the development of the project, e.g. Charles Engel (London), Miriam Friedman Ben-David (Dundee), Colin Coles (Southampton), Sue Baptiste (McMaster-University, Hamilton), Lambert Schuwirth (Maastricht), and Dick Mårtenson (Stockholm).

## 3. Work in progress: ongoing promotion of the reform

The heated debate on the principles of change management marked the phase of implementation and optimisation of the RMC. Essential elements included: thorough consultation, talks with all parties involved (including fireside chats and private talks), teamwork, establishing ownership, shared responsibilities, harnessing committees, and establishing leadership visibility [34], [35]. Thorough consultation was applied to faculty members as well as to external experts. 

An advisory board (AB) was founded to ensure external expertise. Different members of the AB reviewed and evaluated the RMC in 2000, 2002 and in 2005. Experts included: Ann Sefton (Sydney), Charles Engels (London), Dick Mårtenson (Stockholm), and Cees van der Vleuten (Maastricht). Its recommendations affected the next steps and developments of the RMC. While expressing their appreciation for the project as a whole, the experts also provided constructive criticism. Sometimes, they focussed on the curriculum, while another time they critically reviewed the students’ opportunity for self-directed learning and autonomy. At all times, they emphasises the importance of faculty development and participation as well as the impact of the RMC outside of Berlin. In its last evaluation in 2005, the AB stated that the RMC was still too teacher-centred and participation of the whole faculty was not fully implemented yet. Their recommendation for the Charité was to view medical education research a central part of evaluating the curriculum and students’ learning. The AB considered it a necessity to “critically reflect and refine the curriculum in a creative manner.” [41]. In 2002, they saw the latter in danger because of the “large number of necessary tasks for the AG RMC” and they hoped for new structures. Probably because of that observation they appreciated the establishment of a curriculum committee in their last report in 2005, which was installed in 2003 by the Study Committee to “critically evaluate the RMC and work out recommendations for improvements” [42].

The curriculum committee (CoKo) consisted of 16 members and was headed by Walter Burger. Members included professionals from clinical as well as from preclinical disciplines (biochemistry, physiology, medical sociology), members of the AG RMC, and two students. They took up time-consuming work that would last for four years. It resulted in a competence-based catalogue of objectives, which was presented to the faculty in 2005 and was approved as the basis for further planning of the reformed and the traditional curriculum at the Charité. 

Another important quality assurance measure was a systematic internal evaluation of all parts of the curriculum. Questionnaires and discussion groups were used to evaluate the learning progress of students and the motivation among students and teachers. Additionally, evaluation results were compared with evaluation results of the traditional curriculum. Another important evaluation instrument was the Progress Test Medicine (PTM), which was developed at the same time as the RMC [43]. One of the reformers recalls “concluding discussions of the modules and semester-end discussions. I think students have gained trust because we always took them seriously; we always tried to work towards theirs needs – student-centred. Everything was continuously optimised; the pbl concepts were further developed, students had the chance to take entrance tests to assess their learning type, a whole lot. There were many opportunities for counselling in crises that people experienced and so on” [WB, S. 14-15]. Another leadership task was to convince people, to socialise with sponsors and politicians, and to routinely perform “firefighting actions” [WB, S. 12].

## 4. Termination and transformation

With external funding phasing out and following another merging process within the Berlin academic landscape, the end of the RMC emerged. The Charité needed to decide whether to finance the RMC with internal funds or not. That decision-making process lasted from 2005 to 2007, more than two years. Eventually, the notion prevailed that an “expansion of the RMC for all students would not have been possible due to the extensive effort” [44]. Only “a synthesis, a transfer of reform elements in the traditional curriculum” [44] was feasible. That was the birth of the new model curriculum and a compromise to follow the political will to provide the RMC to all students at the Charité – at least formally. At the same time, it was left open which of the “proven parts of the reformed and traditional curriculum” were to be incorporated in a new curriculum. To shed light on the issue, the Dean’s office for student affairs started an exhausting process that lasted from 2007 to 2010. It thus emerged that there were substantial differences with regard to the focus of future teaching and learning and that the above-mentioned change management process had not reached the whole faculty. An in-depth revision of the content and structure of the RMC was not possible due to time constraints or perhaps also due to a lack of willingness. The latter seems more likely, seeing as members of the AG RMC and other supporters of the RMC had been continuously losing ground in the development of the new curriculum. That was the reason why Walter Burger left the Charité, followed by nearly all founders of the RMC. However, pivotal ideas were carried from Berlin to other places in the German-speaking academic landscape. Events and activities like the first Skill Lab Symposium in Berlin in 2007 and the establishment of the Committee ‘‘Communication and Social Competencies’’ by the Association for Medical Education [45] contributed to that dissemination [45]. Many principles of the RMC became visible in the medical curriculum at the Brandenburg Medical School. The RMC does not exist anymore in its previous form and its termination could not have been avoided from today’s point of view. However, it served as a laboratory and experimental field for medical education in German-speaking countries. In addition to students benefitting from the opportunity to experience reformed medical education, this is the value of all model curricula: testing and evaluating the feasibility of educational innovations with small student cohorts, from which many students in traditional curricula will also profit. 

### What has become of the students? 

Eleven cohorts passed the RMC, nearly 700 students. A survey of graduates in 2015 investigated the question of what has become of the former RMC students. An image can be reconstructed based on 164 responses (24%). Their fields of work are highly diverse. Most of the graduates work in Berlin, followed by Brandenburg and North Rhine-Westphalia. Nine students work abroad, four of those in Switzerland. Nearly three-quarter work clinically. Activities in research and teaching are mentioned by 34% reps. 27% of the participants, probably those who work at a university hospital, teaching hospital, or a teaching practice. Most frequent specialty trainings (started and/or completed) were general medicine, anaesthesiology, paediatrics, and neurology. In retrospect, 98% of the responders were very satisfied or satisfied with the RMC and felt well prepared for their career [46]. 

Parts of the RMC were analysed with regard to its effectiveness within several medical education research projects and comparison studies with control groups [27-29]. Key figures from the 2005 final report to the Bund-Länder Commission are listed here as an example. At that time, 316 students started theirs studies within the RMC. Of those, 16 students either dropped out of medicine or switched to the traditional curriculum (5%). Of the RMC first cohort, 18 students (28.5%) took the second part of the state examination (which meant within minimum duration of study), compared with 23.5% in the traditional curriculum. There were no significant differences between the two cohorts in the first years regarding the acquisition of knowledge as tested in the state examination [47]. In 2003, Kiessling et al [28] were able to show that first-year students in the RMC felt more supported and less stressed than students in the traditional track. The so-called KuLM study, a prospective longitudinal survey with first-year and last-year medical students at the Charité, showed that RMC students were more satisfied with their curriculum and experienced lower stress levels than students in the traditional curriculum. Last-year RMC students assessed their competencies with regard of future professional requirements as higher than students in the traditional track. The authors considered the differences regarding communication skills and practical skills particularly drastic, and both competencies were defined as a specific strength of the RMC [48].

The students of the past are the teachers of today and perhaps the decision-makers of tomorrow. We hope that, despite the existing difficult working conditions in the German healthcare system, graduates do remember the basic principles of the RMC, once they find themselves in the position of decision-makers: a “different kind” of medicine with a patient-centred approach, self-determined working, shared decision-making, and learning from others. 

## Acknowledgements

A huge thank you to our interview partners, to Dorothea Eisenmann and Hendrik Bösing for the transfer of the results from the graduates’ survey, Kai Schnabel for critically reviewing the manuscript, and to all companions and supporters of the RMC. 

## Competing interests

The authors declare that they have no competing interests. 
